# TRAF6 triggers *Mycobacterium*-infected host autophagy through Rab7 ubiquitination

**DOI:** 10.1038/s41420-023-01731-4

**Published:** 2023-11-28

**Authors:** Qinmei Ma, Jialin Yu, Li Liu, Xiaoyan Ma, Jiaxue Zhang, Jiamei Zhang, Xiaoping Wang, Guangcun Deng, Xiaoling Wu

**Affiliations:** 1 School of Life Science, Ningxia University, Yinchuan, NingXia 750021 China; 2 Key Lab of Ministry of Education for Protection and Utilization of Special Biological Resources in Western China, Ningxia University, Yinchuan, NingXia 750021 China; 3 The Fourth People’s Hospital of Ningxia Hui Autonomous Region, Yinchuan, NingXia 750021 China

**Keywords:** Autophagy, Infection

## Abstract

Tumor necrosis factor receptor-associated factor 6 (TRAF6) is an E3 ubiquitin ligase that is extensively involved in the autophagy process by interacting with diverse autophagy initiation and autophagosome maturation molecules. However, whether TRAF6 interacts with lysosomal proteins to regulate *Mycobacterium*-induced autophagy has not been completely characterized. Herein, the present study showed that TRAF6 interacted with lysosomal key proteins Rab7 through RING domain which caused Rab7 ubiquitination and subsequently ubiquitinated Rab7 binds to STX17 (syntaxin 17, a SNARE protein that is essential for mature autophagosome), and thus promoted the fusion of autophagosomes and lysosomes. Furthermore, TRAF6 enhanced the initiation and formation of autophagosomes in *Mycobacterium*-induced autophagy in both BMDMs and RAW264.7 cells, as evidenced by autophagic flux, colocalization of LC3 and BCG, autophagy rates, and autophagy-associated protein expression. Noteworthy to mention, TRAF6 deficiency exacerbated lung injury and promoted BCG survival. Taken together, these results identify novel molecular and cellular mechanisms by which TRAF6 positively regulates *Mycobacterium*-induced autophagy.

## Introduction

*Mycobacterium tuberculosis* (Mtb), the pathogenic bacterium responsible for tuberculosis (TB), typically infiltrates the respiratory tract and results in chronic lung lesions [1]. An effective host response against Mtb necessitates the concerted actions of innate and adaptive immune cells, including macrophages, dendritic cells (DCs), neutrophils, and T cells [2, 3]. Macrophages are both the primary host cells and the initial defense against Mtb infection [4–6]. The host cell death pathway represents an efficacious mechanism for eliminating the pathogen during the interaction between macrophages and Mtb [7]. Among them, autophagy is well known for its characteristic structurally diverse and functionally promiscuous for clearance of Mtb [8].

Autophagy-mediated clearance of Mtb is inextricably linked to the formation of autophagosomes and the fusion of autophagosomes with lysosomes [9]. Once Mtb attempts to enter the cytoplasm by disrupting the pathogen-containing vacuoles, cells rapidly generate autophagosomes to sequester Mtb [10]. In the meantime, ubiquitin and galectins recruit the autophagy initiation mechanism to accelerate the formation of mature autophagosomes to fight against pathogen invasions [11]. The regulation of this process is mediated by highly conserved autophagy-related proteins (ATGs), sequestosome 1 (p62), and microtubule-associated protein light chain 3 (LC3) [12]. Notably, a significant quantity of autophagosomes persisted in macrophages infected with the eis-deleted Mtb *H37Rv* strain (Mtb-Δeis), which potentially facilitated Mtb’s establishment of a niche within the host organism [13, 14]. The characteristic acidic environment of lysosomes provides an ideal environment for the activity of lysosomal hydrolase, thereby promoting the degradation of autophagosome contents [15]. Therefore, the fusion of autophagosomes with lysosomes is crucial for Mtb elimination. However, it has been discovered that Mtb-secreted acid phosphatase (SapM) can inhibit the fusion of autophagosomes and lysosomes by targeting host Rab7 [16]. More recently, researchers noted that eukaryotic cells possess the capability to selectively allocate specific cellular materials to the autophagy pathway [17]. This discovery further supports the protective role of autophagy in host anti-Mtb immunity. In fact, as an adjunctive therapy, it has been demonstrated that autophagy plays a contributory role in the effectiveness of frontline anti-TB chemotherapeutics [2, 18, 19]. Therefore, a deeper exploration of autophagy during Mtb infection is imperative for the identification of specific targets to be used in host-directed therapies.

TRAF6 is a member of the TNF-associated factor (TRAF) family and was originally classified as a major mediator of inflammatory responses [20]. In recent decades, intensive investigations of Mtb virulence factors enriched the research information on the ubiquitination of TRAF6. By way of example, PE_PGRS38 inhibited the de-ubiquitination of TRAF6 to regulate the expression of cytokine levels and thereby increase the survival of mycobacteria [21, 22]. PPE68 evades host recognition and dampens host defense responses in the same manner [23]. These findings further support the conclusion that Mtb activates host immune defenses and bacterial immune responses via TRAF6, enabling bacteria to evade or protect themselves from host immunity. Meanwhile, it was found that TRAF6 is involved in the regulation of diverse cellular processes such as development and differentiation, cell cycle progression, and most notably autophagy [24, 25]. As a RING-domain E3 ubiquitin ligase, TRAF6 was reported to rely on lysine 63 (K63)-linked ubiquitin to regulate the occurrence of autophagy [26, 27]. For example, TRAF6 promotes K63-linked ubiquitination of BECN1 to induce Toll-like receptor 4 (TLR4)-mediated autophagy [28]. This finding has also motivated researchers to investigate a new mechanism of TRAF6-BECN1 axis-mediated autophagy. Not surprisingly, multiple proteins involved in autophagosome formation including p62 and AMP-activated protein kinase α (AMPKα), use the TRAF6-BECN1 axis as a springboard to regulate disease progression [29, 30]. However, little is known about the interactions between TRAF6 and proteins involved in the fusion stage of autophagosomes and lysosomes, and whether these interactions can somehow influence the autophagy process. Intriguingly, Chandra et al. mentioned that phagosomes containing virulent Mtb to avoid recruiting Rab7, which inhibits the fusion of phagosomes and lysosomes [31]. This finding brought our attention to a key regulator of the Ras superfamily, Rab7, which is necessary for lysosomal biogenesis, late endosome-lysosome fusion, and autophagosome maturation [32]. This led us to explore the possible interaction of TRAF6 and Rab7 in *Mycobacterium* infection, and further exploration of the role of TRAF6 in *Mycobacterium*-induced fusion of autophagosomes and lysosomes.

Therefore, the purpose of this study was to determine the functional role of TRAF6 in *Mycobacterium*-induced the fusion of autophagosomes with lysosomes. Our results showed that the RING domain of TRAF6 interacted with Rab7 and promoted Rab7 ubiquitination to directly control the association of Rab7 and STX17, thereby promoting the formation of autolysosomes in BCG-infected macrophages. The facilitative effect of TRAF6 in *H37Rv*/BCG-induced autophagy initiation and the formation of autophagosomes was evidenced in RAW264.7 cells and BMDMs. Moreover, cKO-TRAF6 exacerbated BCG-induced lung injury, promoted the survival of bacteria, inhibited CD4+ T cells, and macrophage recruitment. Together, our data demonstrate a novel regulatory mechanism by which TRAF6 positively regulates *Mycobacterium*-induced autophagy, inhibits mouse lung injury and promotes the clearance of BCG.

## Results

### TRAF6 binds to Rab7 via the RING domain

**Fig. 1:**
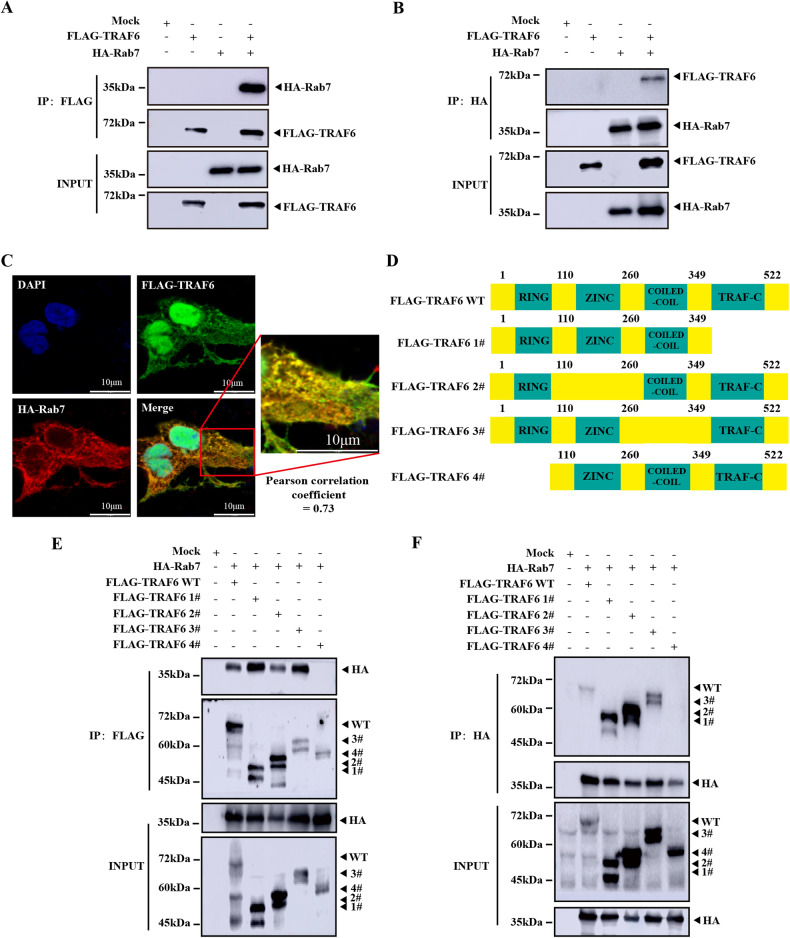
TRAF6 binds to Rab7 via the RING domain. **A**, **B** HEK293T cells were transfected with Mock, 3× FLAG-TRAF6, and 5× HA‐Rab7 as indicated. At 48 h post-transfection, transfected cells were extracted and cell lysates were subjected to immunoprecipitation with anti-FLAG or anti-HA antibody followed by IB using anti-FLAG and anti-HA antibodies. **C** 3× FLAG-TRAF6 was co-transfected with 5× HA‐Rab7 in HEK293T cells, and the colocalization of 5× HA‐Rab7 (Red) and 3× FLAG-TRAF6 (Green) was assessed by immunofluorescence; scale bar: 10 μm. **D** Schematic diagram of TRAF6 truncation mutants. **E** HEK293T cells were transfected with Mock, 3× FLAG-TRAF6 wild type (WT), FLAG-TRAF6 truncated mutants, or 5× HA‐Rab7 as indicated. At 48 h post-transfection, transfected cells were extracted, immunoprecipitated with anti-FLAG antibody, and then subjected to IB assay using anti-FLAG and anti-HA antibodies. **F** HEK293T cells expressing 3× FLAG-TRAF6, FLAG-TRAF6 truncated mutants, or 5× HA-Rab7 were collected and lysed. Then, the cell lysates were immunoprecipitated with anti-HA and then subjected to IB assay using anti-FLAG and anti-HA antibodies. Images were analyzed by ImageJ (Coloc 2 plugin) for colocalization correlation (Pearson correlation coefficient).

Autophagosomes fuse with lysosomes to generate autolysosomes, which are essential for autophagy-dependent intracellular clearance of pathogenic bacteria [33]. Several literatures have reported TRAF6 regulates autophagy progression through interaction with autophagosome maturation-regulating proteins, such as autophagy/beclin-1 regulator-1 (AMBRA1), BECN1, and autophagy-related 9a (ATG9A) [34, 35]. However, there is little known about the interaction between TRAF6 and lysosomal proteins which affects the autophagy process. Rab7 is a small GTPase controlling the trafficking and identity of late lysosomal compartments [36, 37]. To investigate whether TRAF6 interacts with Rab7, 3× FLAG-tagged TRAF6 (FLAG-TRAF6) and 5× HA-tagged Rab7 (HA-Rab7) were co‐transfected into HEK293T cells, and immunoprecipitation (IP) assay was performed with anti-FLAG (for TRAF6) antibody. As shown in Fig. 1A, FLAG-TRAF6 was successfully co-immunoprecipitated with HA-Rab7 (lane 4). The same results were also observed when using anti-HA antibody for IP and anti-FLAG for western blotting (Fig. 1B). Next, we analyzed the intracellular colocalization of TRAF6 and Rab7 in HEK293T cells by using immunofluorescence. As shown in Fig. 1C, the yellow punctate in the merge image indicates that TRAF6 and Rab7 proteins are co-localized in co-transfected HEK293T cells (Pearson correlation coefficient = 0.73). The above results suggested that TRAF6 could binding to Rab7.To further determine the Rab7 interaction site on TRAF6, we truncated TRAF6 into four segments: TRAF6 1# (Δ TRAF-C truncation mutant plasmid), TRAF6 2# (Δ ZINC finger domain truncation mutant plasmid), TRAF6 3# (Δ COILED-COIL truncation mutant plasmid), and TRAF6 4# (Δ RING domain truncation mutant plasmid) (Fig. 1D). HEK293T cells were transfected with Rab7 and TRAF6 wild type (Fig. 1E, lane 2), or different TRAF6 truncation mutants (Fig. 1E, lane 3–6). Then Co-IP assay was performed by using an anti-FLAG antibody. Western blotting analysis showed that the truncation of the RING domain abolished the interaction between TRAF6 and Rab7 (Fig. 1E). The Co-IP experiment using HA antibody as bait also proved this result (Fig. 1F). Collectively, our data indicated that Rab7 binds to TRAF6 via the RING domain.

### *Mycobacterium* infection elevates TRAF6 expression and enhances the binding of TRAF6 and Rab7

**Fig. 2:**
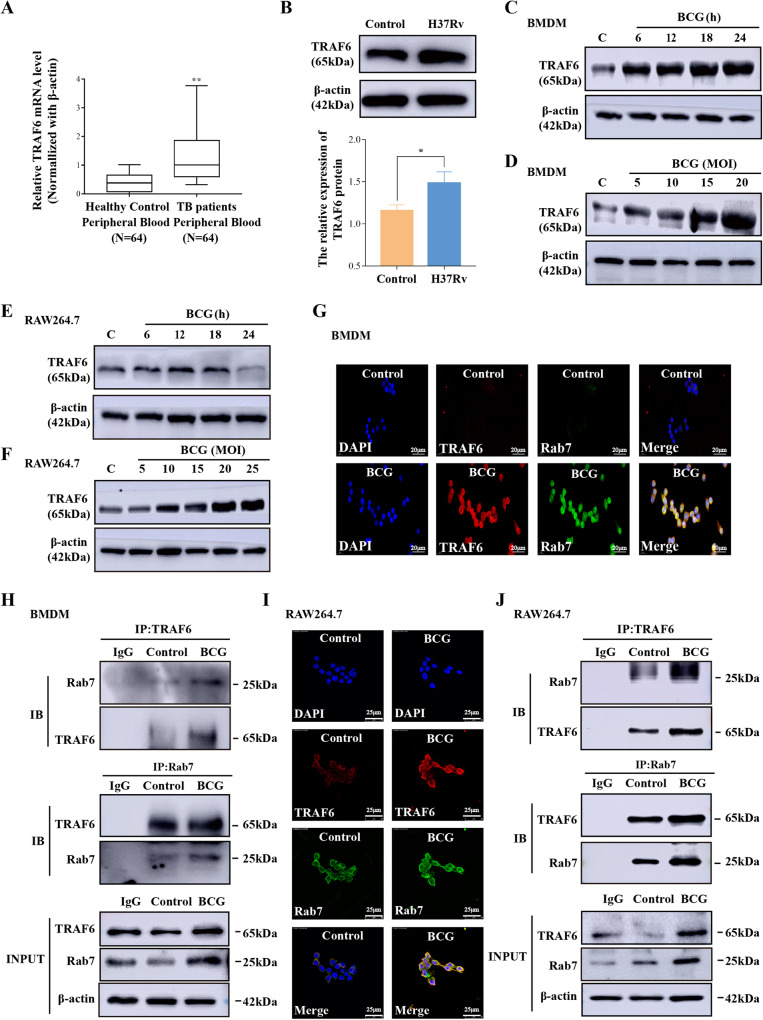
*Mycobacterium* infection elevates TRAF6 expression and enhances the binding of TRAF6 and Rab7. **A** RT-PCR analysis of TRAF6 transcription levels in human peripheral blood from healthy controls (*N* = 64) and active TB patients (*N* = 64). **B** RAW264.7 cells were infected without or with 10 MOI *H37Rv* for 6 h. Cell lysates were immunoblotted with antibodies specific for TRAF6 and β-actin. **C** Western blotting analysis of TRAF6 in BMDMs infected with 5 MOI BCG for 6, 12, 18, and 24 h, respectively. **D** Western blotting analysis of TRAF6 expression in BMDMs infected with 5, 10, 15, and 20 MOI BCG for 6 h. **E** Western blotting analysis of TRAF6 in RAW264.7 cells infected with 10 MOI BCG for 6, 12, 18, and 24 h, respectively. **F** Western blotting analysis of TRAF6 expression in RAW264.7 cells infected with 5, 10, 15, 20, and 25 MOI BCG for 12 h. **G** Immunofluorescence imaging detected co‐localization of TRAF6 (Red) and Rab7 (Green) in BCG-infected BMDMs; scale bar: 20 μm. **H** BMDMs were infected with BCG (MOI = 5) for 6 h. Co-IP assay was performed using lysates with IgG antibody and anti-TRAF6 or Rab7 antibody. IB assay was then performed using anti-TRAF6 and anti-Rab7 antibodies. **I** Rab7 (Green) and TRAF6 (Red) colocalization was detected by immunofluorescence in BCG-infected RAW264.7 cells; scale bar: 25 μm. **J** RAW264.7 cells were infected with BCG (MOI = 10) for 12 h. Co-IP assay was performed using lysates with IgG antibody and anti-TRAF6 or Rab7 antibody. IB assays were then performed using anti-TRAF6 and anti-Rab7 antibodies. The protein ratio was calculated by ImageJ densitometry analysis. The semi-quantitative analysis method of Co-IP refers to the article of Burckhardt et al. [81, 82]. Images were analyzed by ImageJ (Coloc 2 plugin) for colocalization correlation (Pearson correlation coefficient). Relative analysis results are described in Supplementary Fig. 1. Data were shown as the mean ± SEM, and one representative experiment from three independent experiments is shown. *p < 0.05, **p < 0.01.

Recent reports have suggested that TRAF6 has several different functions in infectious diseases [38, 39]. To explore the role of TRAF6 during *Mycobacterium* infection, we first examined the expression of TRAF6 in human peripheral blood from healthy people and active TB patients. The results demonstrated that compared with healthy people, the expression of TRAF6 mRNA was upregulated in human peripheral blood from active TB patients (Fig. 2A). To further confirm the effect of *Mycobacterium* infection on TRAF6 expression, macrophages were infected with *Mycobacterium* BCG or *H37Rv*. The infection efficiency was confirmed by using 5-Carboxyfluorescein diacetate N-succinimidyl ester dye. The observation results showed that every cell in the field of view was infected with green fluorescence marked *Mycobacterium* (Supplementary Fig. 1A, B). At the same time, we used flow cytometry to detect the green fluorescence intensity and carrying ratio in BMDMs. The results show in Supplementary Fig. 1C, 95% of the cells were infected with green fluorescence marked *Mycobacterium*. On this basis, we performed western blotting to detect the expression of TRAF6 under different *Mycobacterium* infection conditions. The results showed that *H37Rv* infection elevated TRAF6 expression in RAW264.7 cells (Fig. 2B). Meanwhile, BCG infection upregulated the expression of TRAF6 in BMDMs and differences were significant at 5 MOI and 6 h (Fig. 2C, D and Supplementary Fig. 1D, E). The same experiment was carried out in RAW264.7 cells. As shown in Fig. 2E, F and Supplementary Fig. 1H, I, RAW264.7 cells infected with BCG at 10 MOI for 12 h presented a higher differential expression of TRAF6 than that in un-infected cells. Similar results were confirmed by RT-PCR (Supplementary Figs. 1F, G, J, K). Together, the above results indicated that *Mycobacterium* infection upregulated the expression of TRAF6 both in vivo and in vitro. To assess the binding ability of TRAF6 and Rab7 during *Mycobacterium* infection, we first determined the colocalization of TRAF6 and Rab7 in BCG-infected BMDMs. As shown in Fig. 2G and Supplementary Fig. 1L, the colocalization of TRAF6 and Rab7 was significantly increased in the BCG infection group than that in the control group (*p* < 0.001, Pearson correlation coefficient 0.87 vs 0.331). Similar results were also observed in RAW2647 cells (Fig. 2I and Supplementary Fig. 1O). Meanwhile, the endogenous-cellular Co-IP experiments were performed to detect the interaction between TRAF6 and Rab7 in BMDMs. We found the binding efficiency of TRAF6 to Rab7 was higher after BCG infection (Fig. 2H and Supplementary Fig. 1M, N). These results were also confirmed in RAW264.7 cells (Fig. 2J and Supplementary Fig. 1P, Q).

### TRAF6 strengthens the binding ability of Rab7 and STX17 by promoting Rab7 ubiquitination in *Mycobacterium*-infected macrophages

**Fig. 3:**
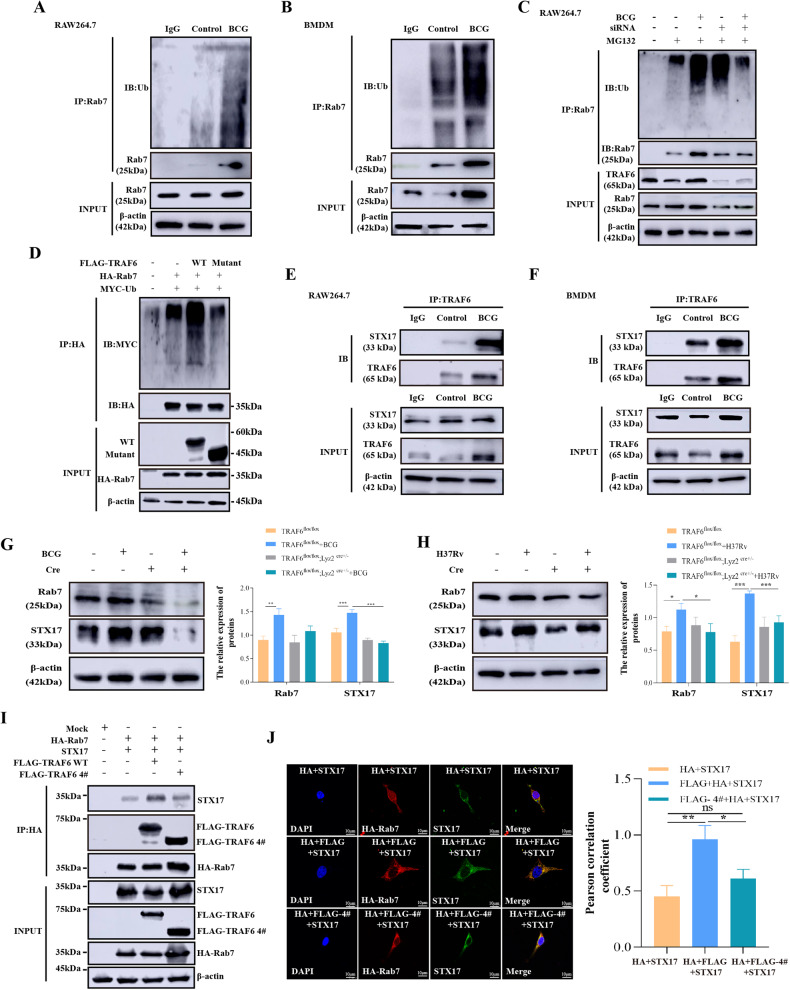
TRAF6 strengthens the binding ability between Rab7 and STX17 by promoting Rab7 ubiquitination in *Mycobacterium*-infected macrophages. **A** IP assay analysis of Rab7 ubiquitination in RAW264.7 cells with or without BCG infection (MOI = 10) for 12 h and treated with MG132 (10 μM) for 6 h before harvest. IB assays were performed using anti-Rab7 and anti-Ub antibodies. **B** BMDMs were treated with or without BCG infection (MOI = 5) for 6 h and treated with MG132 (10 μM) for 6 h before harvest. IP assay was performed using lysates with anti-Rab7. IB assay was performed using anti-Rab7 and anti-Ub antibodies. **C** IP assay was performed with anti-Rab7 antibody using RAW264.7 cells transfected with *siRNA-1810* under the BCG-infection condition and treated with MG132 (10 μM) for 6 h before harvest. IB assay was performed with anti-Rab7 and anti-Ub antibodies. **D** HEK293T cells were transfected with Mock, 3× FLAG-TRAF6, FLAG-TRAF6 truncated mutants, 5× HA‐Rab7, MYC-Ub as indicated. At 48 h post-transfection, transfected cells were extracted and cell lysates were subjected to immunoprecipitation with anti-HA antibody followed by IB using anti-MYC and anti-HA antibodies. **E**, **F** RAW264.7 (**E**) and BMDMs (**F**) were used to examine the interaction of TRAF6 and STX17 after BCG infection. **G** The expression of Rab7 and STX17 was detected by western blotting in BCG-infected BMDMs. **H** The expression of Rab7 and STX17 was detected by western blotting in *H37Rv*-infected BMDMs. **I** The lysates from HEK293T cells overexpressing STX17, 5× HA‐Rab7, FLAG-TRAF6 truncated mutants, and 3× FLAG-TRAF6 were immunoprecipitated with anti-HA and immunoblotted using the indicated antibodies. **J** 3× FLAG-TRAF6, FLAG-TRAF6 truncated mutants, STX17 were co-transfected with 5× HA‐Rab7 in HEK293T cells, and the colocalization of 5× HA‐Rab7 (Red) and STX17 (Green) was assessed by immunofluorescence. Scale bar: 10 μm. The protein ratio was calculated by ImageJ densitometry analysis. The semi-quantitative analysis method of Co-IP refers to the article of Burckhardt et al. [81, 82]. Images were analyzed by ImageJ (Coloc 2 plugin) for colocalization correlation (Pearson correlation coefficient). Relative analysis results are described in Supplementary Fig. 2. Data were shown as the mean ± SEM, and one representative experiment from three independent experiments is shown. *p < 0.05; **p < 0.01; ***p < 0.001.

TRAF6 serves as a K63-linked E3 ubiquitin ligase by interacting with numerous endogenous proteins to regulate cell cycle progression or signaling transduction processes [27, 40–43], we hypothesized that TRAF6 might regulate BCG-induced autophagy by ubiquitinating Rab7. To confirm this hypothesis, we detected the ubiquitination of Rab7 in BCG-infected macrophages. As shown in Fig. 3A, B and Supplementary Fig. 2A, B, BCG induced Rab7 ubiquitination in macrophages. Meanwhile, *siRNA-TRAF6* greatly inhibited BCG-induced Rab7 ubiquitination and protein expression level (Fig. 3C and Supplementary Fig. 2C). This further proves that TRAF6 promotes the occurrence of autophagy by exerting its own K63 ubiquitination function. Again, we transferred HA-Rab7, MYC-Ub, FLAG-TRAF6, or TRAF6 4# (Δ RING domain truncation mutant plasmid) to HEK293T cells, and IP assay was performed by using anti-HA antibody. As shown in Fig. 3D and Supplementary Fig. 2D, TRAF6 promoted the ubiquitination level of Rab7. However, when the RING domain of TRAF6 was mutated, the ubiquitination level of Rab7 was significantly reduced. Meanwhile, there were no significant differences between the Rab7 + Ub co-transfection group and Rab7 + Ub + TRAF6 mutant plasmid co-transfection group. This finding reflected that TRAF6 lost the ability to ubiquitinate Rab7 when the interaction was abolished. These results indicate that the RING domain of TRAF6 is not only essential for the interaction but also ubiquitination of Rab7.

Numerous studies have shown that autophagosomes directly fuse with Rab7-positive lysosomes, and the interaction between Rab7 and STX17 (a key autophagosome protein) is essential for this process [44]. Considering that TRAF6 ubiquitinated Rab7 which may further regulate the fusion of autophagosomes with lysosomes by forming Rab7-STX17 complex, we utilized Co-IP experiments to evaluate whether there is also a relationship between TRAF6 and STX17 in BCG-infected macrophages. The results indicated that TRAF6 interacted with STX17 and BCG infection to strengthen their binding ability (Fig. 3E, F and Supplementary Fig. 2E, F). In addition, we investigated the effect of TRAF6 on the expression of Rab7 and STX17 in *Mycobacterium*-infected BMDMs. As shown in Fig. 3G, H, cKO-TRAF6 suppressed the expression of Rab7 and STX17 in *Mycobacterium*-infected macrophages. Subsequently, HA-Rab7, STX17, and FLAG-TRAF6 or TRAF6 4# were expressed in HEK293T cells, and an IP assay was performed by using an anti-HA antibody. As shown in Fig. 3I and Supplementary Fig. 2G, TRAF6 promoted the binding ability of STX17 to Rab7, but TRAF6 RING domain deletion mutant inhibited this phenomenon. The colocalization detection via immunofluorescence displayed a similar result (Fig. 3J). Based on the above findings, it can be concluded that TRAF6 promotes Rab7 binding to STX17 by ubiquitinating Rab7 in *Mycobacterium*-infected macrophages.

### TRAF6 contributes to *Mycobacterium*-mediated autophagosome initiation and formation

**Fig. 4:**
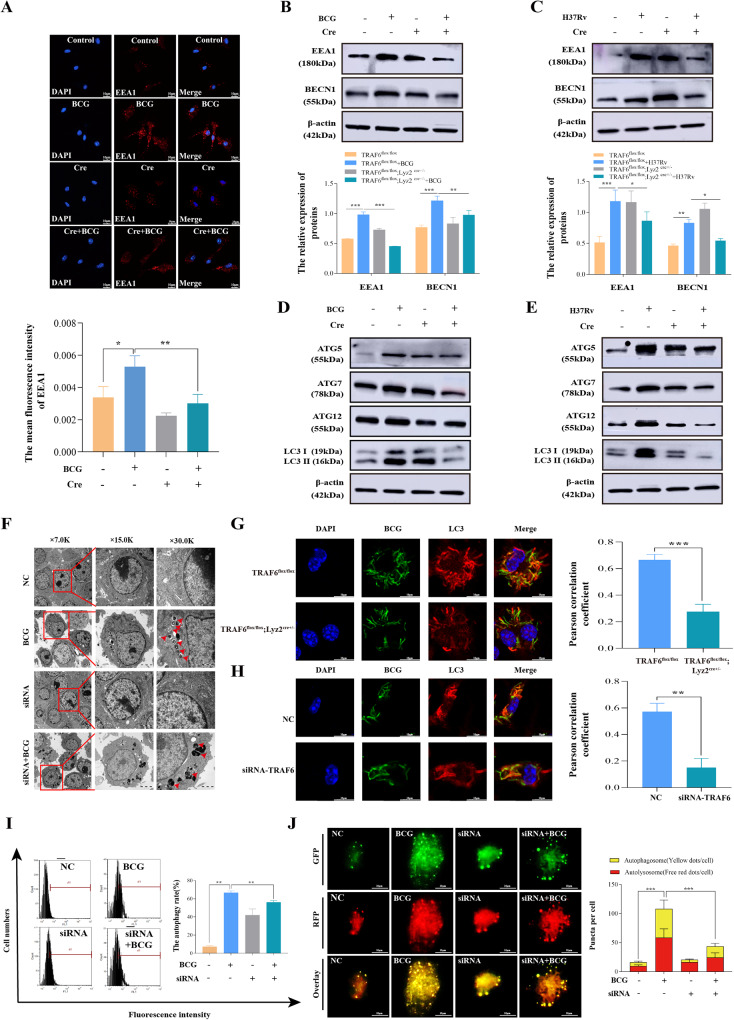
TRAF6 contributes to *Mycobacterium*-mediated autophagosome initiation and formation. **A** Representative fluorescence microscopy images of BMDMs infected with BCG. The red fluorescence intensity indicated the level of intracellular EEA1. Scale bar: 10 μm. Bar graph shows the mean fluorescence intensity. **B** The expression of EEA1 and BECN1 in BCG-infected BMDMs was detected by western blotting. Lower: quantification of EEA1 and BECN1 protein expression in BCG-infected BMDMs. **C** The expression of EEA1 and BECN1 in *H37Rv*-infected BMDMs was detected by western blotting. Lower: quantification of EEA1 and BECN1 protein expression in *H37Rv*-infected BMDMs. **D**, **E** Protein levels of ATG5, ATG7, ATG12, and LC3 in BCG- (**D**) and *H37Rv* (**E**) -infected BMDMs. **F** After incubation with *siRNA-1810* and treatment with BCG for 12 h, the autophagosome-like structures were observed by TEM. The red arrow indicates the autophagosome-like structures. Asterisks represent the BCG. **G**, **H** Confocal microscopy analysis for colocalizations of BCG with LC3 in BMDMs (**G**) or RAW264.7 cells (**H**) was then immunostained using an anti-LC3 antibody (Red). BCG (Green) was stained with 5-Carboxyfluorescein diacetate N-succinimidyl ester 30 min before infection. Scale bars, 10 µm. **I** Cyto-ID^®^ autophagy detection kit was used to measure the autophagy rate in RAW264.7 cells, and the relative expression is shown in bar diagrams. **J** Representative images of autophagic flux puncta in RAW264.7 cells were captured by confocal laser microscopy; yellow puncta indicate autophagosomes and red dots indicate autolysosomes; scale bar: 5 μm. Quantitative analysis of autophagosomes and autolysosomes per cell. The protein ratio was calculated by ImageJ densitometry analysis. Images were analyzed by ImageJ (Coloc 2 plugin) for colocalization correlation (Pearson correlation coefficient). Relative analysis results are described in Supplementary Fig. 3. Data were shown as the mean ± SEM, and one representative experiment from three independent experiments is shown. *p < 0.05; **p < 0.01; ***p < 0.001.

Notably, Rab7 localizes in the lysosomal and STX17 localizes to the outer membrane of completed autophagosomes, the combination between them that represented the formation of autolysosomes [45]. Meanwhile, our data showed that TRAF6 affects Rab7 binding to STX17 by ubiquitinating Rab7, so we wanted to further explore whether TRAF6 is involved in regulating the formation of autolysosomes. Early endosomal antigen 1 (EEA1) and BECN1 are required for autophagosome formation [46, 47]. We first examined the effect of cKO-TRAF6 on EEA1 expression in BCG-infected BMDMs using immunofluorescence. As shown in Fig. 4A, EEA1 was highly expressed in BMDMs after BCG infection, but cKO-TRAF6 suppressed the expression of EEA1. Additionally, western blotting results showed that EEA1 and BECN1 were upregulated in both BCG- and *H37Rv*-infected BMDMs, but cKO-TRAF6 downregulated EEA1 and BECN1 expression (Fig. 4B, C). Autophagy-related proteins mediate the formation of autophagosomes [48]. Western blotting was performed to measure the ATG5, ATG7, ATG12, and LC3 protein expression. The data are displayed in Fig. 4D, E and Supplementary Fig. 3A, B. The expression of ATG5, ATG7, ATG12, and LC3 in BMDMs was remarkably enhanced after both BCG (Fig. 4D and Supplementary Fig. 3A) and *H37Rv* (Fig. 4E and Supplementary Fig. 3B) infection. However, cKO-TRAF6 significantly reduced the expression of ATG5, ATG7, ATG12, and LC3 in BCG- and *H37Rv*-infected BMDMs. All the above results suggested that TRAF6 regulates the expression of autophagosome key proteins in *Mycobacterium*-infected macrophages.

Since TRAF6 was increased in RAW264.7 macrophages upon BCG infection, small interfering RNA was used to detect the effects of TRAF6 knockdown on autophagosome formation, colocalization of LC3 and BCG, autophagic flux and autophagy rate in BCG-infected macrophages. Three small interfering RNAs targeting TRAF6 (*siRNA-1153*, *siRNA-1810*, and *siRNA-4524*) were designed, and the knockdown efficiency was verified by western blotting. As shown in Supplementary Fig. 3C, D, *siRNA-1810* decreased the expression of TRAF6 in RAW264.7 cells with or without BCG. Therefore, *siRNA-1810* was used in subsequent experiments. Transmission electron microscopy (TEM) showed that there were significantly more autophagosome-like structures encased BCG (black matter) in the BCG infection group compared with the *TRAF6 siRNA-1810* and BCG co-treatment group (Fig. 4F and Supplementary Fig. 3E). Further experiments demonstrated that both cKO-TRAF6 (Fig. 4G) or *TRAF6 siRNA* (Fig. 4H) reduced the colocalization of BCG with LC3 in macrophages. Next, we employed a Cyto-ID^®^ autophagy detection kit to detect autophagy rate with flow cytometry. The results showed that the autophagy rate in the BCG-infected group was markedly increased compared to that in the *NC siRNA* group, and TRAF6 knockdown attenuated the BCG-induced RAW264.7 cell autophagy rate (Fig. 4I). We also monitored autophagic flux by using RFP-GFP adenovirus. As shown in Fig. 4J, *TRAF6 siRNA-1810* significantly reduced both red and yellow fluorescent spots in BCG-infected RAW264.7 macrophages. Overall, we concluded that TRAF6 positively regulates autophagy initiation and autophagosome formation in *Mycobacterium*-infected macrophages.

### TRAF6 suppresses BCG survival by activating autophagy and recruiting macrophages, CD4+ T cells

**Fig. 5:**
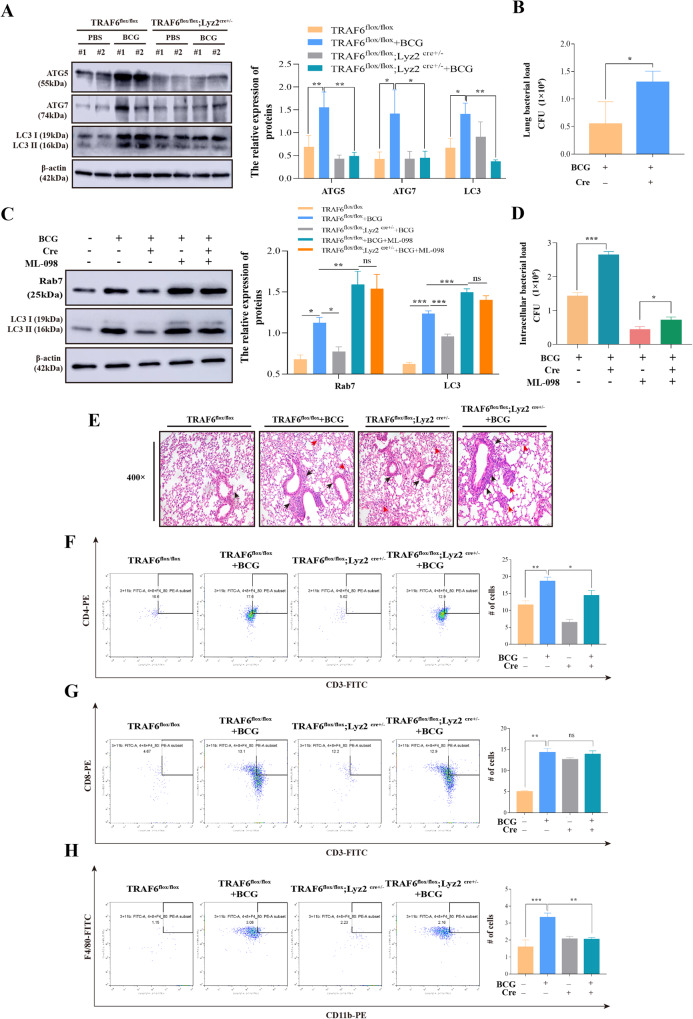
TRAF6 suppresses BCG survival by activating autophagy and recruiting macrophages, CD4+ T cells. **A** Western blotting was performed to detect the expression of ATG5, ATG7, and LC3 in mouse lung tissue. *N* = 5 in each group. Right: quantitative analyses of those target proteins. **B** Effect of cKO-TRAF6 on bacterial load in mouse lung tissue. Mice were infected with BCG (CFU = 2 × 10^6^) for 21 days and then lysed, diluted 1000-fold before use, and cultured in Middlebrook 7H10 agar for 21 days to detect bacterial loads (*N* = 3). **C** BMDMs were treated with ML-098 (0.5 μM/L) for 3 h and infected with BCG for 6 h after changing the culture medium. The expression of Rab7 and LC3 was determined by western blotting. Right: quantitative analyses of the relative protein expression of Rab7 and LC3. **D** Effects of TRAF6 on intracellular bacterial growth after ML-098 treatment (*N* = 3). A total of 1 × 10^6^ cells/well were placed on a 6-well plate incubated for 12 h and infected with BCG at 5 MOI for 6 h. BCG growth was assessed by CFU assay. **E** The lung tissues of mice infected with BCG were collected and stained with H&E, (magnification, 400x). Black arrows indicate inflammatory cell infiltration and the red arrows show interalveolar septal thickening and alveolar edema. **F**–**H** The bronchoalveolar lavage fluid was prepared after airway perfusion with BCG, CD4+ T cells (**F**), CD8+ T cells (**G**), and macrophage (**H**) recruitment were examined by flow cytometry. Quantification of the immune cells is shown on the right side of the flow cytometry image. *N* = 5 in each group. The protein ratio was calculated by ImageJ densitometry analysis. Data were shown as the mean ± SEM, and one representative experiment from three independent experiments is shown. *p < 0.05; **p < 0.01; ***p < 0.001.

Autophagy is critically associated with bacterial elimination in infectious diseases including TB [49, 50]. Interestingly, there is much evidence indicating that TRAF6 is functionally implicated in bactericidal activity [51, 52]. We therefore investigated whether TRAF6 was functionally associated with bactericidal activity by regulating autophagy. We first detected the effect of cKO-TRAF6 on the expression of autophagy-related proteins in mouse lung tissue. As shown in Fig. 5A, compared with the control group, the expression of ATG5, ATG7, and LC3 was significantly upregulated in BCG infection. However, cKO-TRAF6 suppressed the expression of ATG5, ATG7, and LC3 in mouse lung tissue. This is consistent with the results of the in vitro assay. Subsequently, to determine the role of TRAF6 in regulating lung bactericidal survival, we used the plate coating method to detect the changes in bacterial load in mouse lungs. As shown in Fig. 5B, compared with that in the BCG infection group, the bacterial load was significantly upregulated in the cKO-TRAF6 + BCG group. To further certify whether TRAF6 enhances BCG clearance through the autophagosome-lysosomal pathway, BMDMs were treated with a Rab7 activator (ML-098) prior to BCG infection. From the data in Fig. 5C, compared with the untreated group, ML-098 increased the expression of autophagy-related proteins Rab7 and LC3 in BCG-infected BMDMs. However, there were no significant differences in the expression of Rab7 and LC3 between the BCG + ML-098 and cKO-TRAF6 + BCG + ML-098 groups. Meanwhile, the results of a bacterial loads assay demonstrate that cKO-TRAF6 extremely significantly increased intracellular bacterial load. However, after treated with ML-098, although cKO-TRAF6 significantly increased the intracellular bacterial load, compared with the cKO-TRAF6 + BCG group, the upward trend of bacterial load was attenuated after Rab7 activator treatment (Fig. 5D). The above data indicate that TRAF6-mediated clearance of BCG partially relies upon the fusion of autophagosomes with lysosomes.

Lung histopathology was observed to explore the effects of cKO-TRAF6 on BCG-induced lung injury. H&E staining showed a massive accumulation of infiltrated cells, reduction of alveolar septa, and the alveolar wall thickened in the BCG-infected TRAF6^flox/flox^ group, while these pathological changes were enhanced in the TRAF6^flox/flox;^ Lyz2^cre+/-^ + BCG group (Fig. 5E). The literature indicates that caused by Mtb infection involves the recruitment of immune cells [53, 54]. Therefore, we examined the recruitment of macrophages, CD4+ T cells, and CD8+ T cells 21 days after BCG infection. As shown in Fig. 5F–H, compared with the PBS-treated (control) group, BCG infection elicited macrophage, CD4+ T cells, and CD8+ T cells recruitment, cKO-TRAF6 inhibited macrophage and CD4+ T cells recruitment by BCG infection. However, there was no significant difference in the recruitment of CD8+ T cells between the TRAF6^flox/flox;^ Lyz2^cre+/-^ + BCG group, and the BCG alone infection group. The above studies demonstrated that TRAF6 deficiency exacerbates lung injury in BCG infection.

## Discussion

The interaction between host autophagy responses and Mtb is a complex phenomenon that yields disparate outcomes, ranging from the elimination of bacteria to the establishment of latent infection [55]. This process involves many factors, including immune cells, inflammatory mediators, and many enzymes [56–58]. As a representative example, cytosolic Mtb DNA is recognized by the cGAS-STING signaling pathway, leading to the labeling of Mtb by ubiquitinated proteins [59]. This labeling, along with other factors, is then identified by sequestosome 1/p62-like receptors (SLRs), a subset of autophagy-related receptors involved in antimicrobial defense [60]. The SLRs recognize the ubiquitinated Mtb and then direct them to autophagosomes for subsequent degradation [61]. The expression of scavenger receptors (SRs) on macrophages and mycobacterial antigen presentation are also involved in the autophagic anti-Mtb mechanism [62]. The peptides produced from autophagy can generally be presented by MHC-II molecules, making the autophagy pathway an essential origin of antigens for CD4+ T cells [63, 64]. However, Mtb successfully combats macrophage microbicidal mechanisms by inhibiting the submission of its antigen by MHC-II molecules. Recent evidence has confirmed that Mtb could abolish the activation of MAPK and NF-κB which were two critical signaling pathways in resisting Mtb infection by targeting TRAF6 [65]. Meanwhile, it is well documented that Mtb virulence factors enhance their intracellular survival through the ubiquitin ligase function of TRAF6, suggesting that TRAF6 is a key molecule for the entire immune system [66]. It is also for this reason that TRAF6 was quickly studied in autophagy-related fields. For example, peroxiredoxin 1 (PRDX1) interacts with the RING domain of TRAF6 and negatively regulates TLR4 signaling for NF-κB activation and autophagy [27]. The same results were also found in chronic inflammatory pain [67]. VP3, an avian virus, utilizes the mechanism of inhibiting TRAF6-mediated NF-κB activation to evade the host’s innate immunity. This is achieved through the induction of TRAF6 autophagic degradation in a p62-dependent manner [68]. However, no previous literature is available with regard to the role of TRAF6 in Mtb-induced autophagy. In our study, we found that *Mycobacterium* upregulated the expression of TRAF6 in vitro and in vivo and cKO-TRAF6 inhibited *Mycobacterium*-induced autophagy. Further analysis revealed that TRAF6 mediates the fusion of autophagosomes and lysosomes to inhibit the survival of BCG by ubiquitinating the key lysosomal protein Rab7. Our study may provide an advanced understanding of the anti-mycobacterial mechanisms of autophagy.

As a fundamental bridge in various signaling pathways, TRAF6 is crucial to maintaining cellular homeostasis caused by pathogen infection [22]. Klink et al. found that Mtb decreased TRAF6 protein levels while greatly enhancing TRAF6 mRNA levels in peritoneal mouse macrophages after a prolonged infection of 48 h [69]. However, our present study showed that TRAF6 protein levels were significantly upregulated in *H37Rv*-infected RAW264.7 cells. This is likely due to the differences in MOI and time of Mtb infection. Meanwhile, we found that the infection time and MOI of TRAF6 differentially expression is different in BCG-infected RAW264.7 cells and BMDMs, which indicates that the expression of the same protein in different cell lines is widely different. Recent studies have demonstrated that the elevated cytosolic TRAF6 levels in Mtb-infected macrophages may suppress Mtb survival, which is in line with the results of our study. More importantly, our results indicated that TRAF6 clears bacteria in part by mediating the fusion of autophagosomes with lysosomes. The successful establishment of Mtb infection is known to be largely dependent on its early interactions with host innate immune cells which recognize and uptake Mtb through various pattern recognition receptors (PRRs) [2]. Several publications demonstrated that TRAF6 is critical for the activation, differentiation, and survival of T cells, B cells, and macrophages [26, 70, 71]. We found that macrophages and CD4+ T cells increased significantly in the mice lungs after BCG instillation, indicating that these immune cells were rapidly recruited to the lung tissue in BCG infection. However, cKO-TRAF6 inhibited the recruitment of CD4+ T cells and macrophages. This phenomenon illustrates that TRAF6 contributes to be the main contributing factor for BCG-induced recruitment of immune effector cells into the lungs. Autophagy cooperates with innate immunity to clear the intracellular bacteria [72]. Our data suggest that TRAF6 positively regulates autophagy and the inhibition of autophagy levels caused by cKO-TRAF6 aggravates BCG-induced lung injury. The empirical findings in this study provide a new understanding of TRAF6 in host-bacterial interactions during *Mycobacterium* infection and further explain the ability of autophagy to resist pathogenic bacteria.

TRAF6, acting as a bridging protein, can interact with other proteins through its RING finger domain and exhibits K63-linked E3 ubiquitin ligase activity. The interaction between TRAF6 and BECN1 is critical for TLR-induced autophagy activation and is functionally implicated in the evolution of various diseases. Upon TLR stimulation, a lysine located at the BH3 (Bcl-2 homology 3) domain of BECN1 serves as the main site for TRAF6-mediated K63-linked ubiquitination, leading to the activation of autophagy [28]. However, BECN1 in DAMP-treated alveolar macrophages suppressed K63-linked ubiquitination of TRAF6 [73]. This indicates that there is a bidirectional regulatory relationship between TRAF6 and BECN1. The present study also found that TRAF6 promoted the ubiquitination of Rab7 in BCG-infected macrophages. LC3 and p62 are involved in autophagosome maturation. The study indicated that TRAF6 promoted the recognition and selective autophagic degradation of cis-acting circRNA generated by β-catenin (CTNNB1) by interacting with LC3 in colorectal cancer cell lines [74]. Confirmation of Rab7 is important not only to morphology and turnover of lysosomes but also in autolysosome fusion [75]. Many related studies have reported that Rab7 determines the fusion specificity of autolysosomes by interacting with SNARE proteins [76]. A few effectors were reported to participate in Rab7- and SNAREs-mediated fusion of lysosomes with autophagosomes, such as PLEKHM1 and PG5 [77, 78]. Although EPG5 functions as a tethering factor that is limited to autophagosome-lysosome fusion, it regulates Rab7 to determine the fusion specificity of autolysosomes and late endosomes by interacting with SNARE proteins [76]. STX17, a SNARE protein resident in the endoplasmic reticulum, serves as an effector of Rab7 and plays a crucial role in the fusion of autophagosomes with lysosomes. Interestingly, upon induction of autophagy starvation, STX17 was observed to translocate to the ER-mitochondria contact site to initiate the formation of phagophores, supporting a positive role of STX17 in the early step of autophagy [45, 79]. This may be due to differences in macro-autophagy and mitophagy. There are also many studies on the relationship between Rab7 and Mtb infection, but they only stay at Rab7 controls lipid droplet-phagosome association during mycobacterial infection [80]. In our study, we found that the RING domain of TRAF6 ubiquitinates Rab7 to promote the binding of Rab7 and STX17 which further promotes autophagosome and lysosome fusion in BCG-infected macrophages. Our results provided basic information for further study of TRAF6 as well as further enriched the function research of TRAF6 regulates Mtb-induced autophagy.

**Fig. 6:**
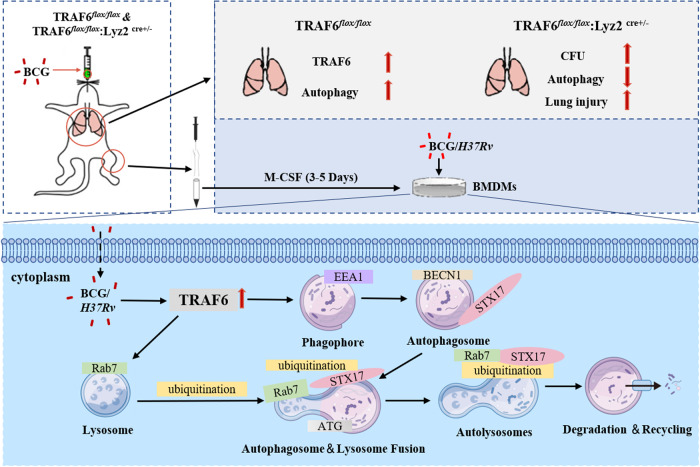
TRAF6 promotes the fusion of lysosomes and autophagosomes through ubiquitination of Rab7 and enhances macrophage bacterial clearance ability. Proposed model for the functional mechanism by which TRAF6 regulates *Mycobacterium*-induced macrophage autophagy. In vivo study, the pulmonary infection of BCG promoted the expression of TRAF6 which was accompanied by the occurrence of autophagy in mouse lung tissue. Further investigation suggested that TRAF6 promoted autophagy and weakened bacteria survival. In vitro, TRAF6 ubiquitinated the key lysosomal protein Rab7 which promoted its ability in recruiting STX17 to lysosomes in BCG-infected macrophages. This led to the fusion of autophagosomes and lysosomes and facilitated bacterial clearance.

In conclusion, TRAF6 positively regulates the autophagosome-lysosome fusion to promote BCG clearance and inhibit mouse lung injury. Regarding the regulatory mechanisms by which TRAF6 regulates the fusion of autophagosome and lysosome, we found that TRAF6 ubiquitinated Rab7 to promote the binding of Rab7 and STX17 in BCG-infected macrophages (Fig. 6). To sum up, we concluded that TRAF6 participates in pathogen-host interactions by regulating the fusion of autophagosomes with lysosomes during autophagy. In addition, we considered that TRAF6 also plays an important positive role in the initiation stage of autophagy and the maturation stage of autophagosomes, which is worthy of further exploration. Our current results will help us to better understand the relationship between the pathogen and the host, and thus the pathogenicity of bacteria.

## Materials and methods

### Antibodies and reagents

Antibodies were used at a dilution of 1:1000 unless otherwise specified. Rabbit monoclonal antibodies against mouse TRAF6 (40675), and EEA1 (109110) were purchased from Abcam (Cambridge, UK). Rabbit monoclonal antibodies against mouse ATG5 (D5F5U), ATG7 (D12B11), ATG12 (D88H11), Ubiquitin (3936), HA-Tag (3724), FLAG-Tag (14793), and Rab7 (9367) were obtained from Cell Signaling Technology (Boston, USA). Rabbit polyclonal antibodies against mouse LC3 (14600-1-AP), and STX17 (17815-1-AP), and the mouse monoclonal antibodies against mouse MYC-Tag (60003-2-Ig), FLAG-Tag (20543-1-AP), BECN1 (66665-1-Ig) and HA-Tag (66006-2-Ig) were purchased from Proteintech (California, USA). Mouse monoclonal antibodies against mouse Rab7 (sc-376362) and TRAF6 (sc-8409) were obtained from Santa Cruz Biotechnology (Texas, USA). Additionally, anti-rabbit IgG (7074) and anti-mouse IgG (7076) were purchased from Cell Signaling Technology (Boston, USA). HRP-conjugated goat anti-rabbit IgG (SA00001-2), and HRP-conjugated goat anti-mouse IgG (SA00001-1) were obtained from Proteintech (Chicago, USA). Mouse anti-rabbit IgG HRP (M21006) and goat anti-rabbit IgG HRP (M21007) were purchased from Abmart (Shanghai, China). Goat anti-mouse/rabbit IgG secondary antibody conjugated to Alexa Fluor 555 (A32727, A21428) and goat anti-rabbit/mouse IgG secondary antibody conjugated to 488 (A11008, A11001) were purchased from Invitrogen (Massachusetts, USA). Other immunofluorescence reagents were purchased from ZSGB-Bio (Beijing, China). The proteasome inhibitor MG132 (133407-82-6), Rab7 activator ML-098 (HY-19800), and 5-Carboxyfluorescein diacetate N-succinimidyl ester (HY-D0056) were purchased from MedChemExpress (Shanghai, China). Actin-Tracker Red-555 (C2203S) was obtained from Beyotime (Shanghai, China).

### Clinical sample collection

The research protocols were approved by the Fourth People’s Hospital of Ningxia Hui Autonomous Region (Yinchuan, China). All clinical investigations were accomplished following the principles expressed in the Declaration of Helsinki (DoH) and all patients provided written informed consent to participate and publish. Active TB patients (*N* = 64; mean age = 52.89 ± 19.35) and Healthy control (*N* = 64; mean age = 43.71 ± 12.63) were collected from June 2020 to December 2020. Blood samples from 128 cases were detected with the reverse transcription-polymerase chain reaction (RT-PCR). The correlation of TRAF6 expression was estimated in whole blood of active TB patients by GraphPad Prism version 7.0 analysis. Inclusion criteria for active TB patients: The diagnosis of active TB patients was based on the Chinese Medical Association guidelines for the primary diagnosis and treatment of tuberculosis (2018) and the guidelines for the diagnosis and treatment of tuberculosis (2001). The criteria for active TB patients included in the study included the following: (1) Mtb was detected in the sputum of the patients or Mtb was positive in vitro culture; (2) the sputum smears of the patients were negative, but tuberculosis was confirmed by imaging, clinical symptoms, and related examinations. The exclusion criteria for active TB patients were as follows: (1) patients suffering from immunodeficiency diseases or other immune system diseases; (2) patients with lung cancer, chronic obstructive pulmonary disease, or other lung diseases; (3) patients infected by hepatitis B virus or other bacteria and viruses; and (4) patient with tuberculosis in other parts, heart disease or tissue and organ failure.

### Mice

All mouse strains were maintained on a C57BL/6J background and were grown at 22 ± 1 °C in a specific pathogen-free (SPF) research facility with a 12 h light/dark cycle at Ningxia University (Yinchuan, Ningxia, China). All animal experiments were approved by the animal ethics committees of Ningxia University and were performed according to institutional guidelines (No. NXU-IACUC-2022092701, 27 September 2022). TRAF6^flox/flox^ mice and TRAF6^flox/flox^; Lyz2^cre+/-^ mice were purchased from Shanghai Biomodel Organism Science & Technology Development (Shanghai, China). TRAF6^flox/flox^; Lyz2^cre+/-^ mice were generated by crossing TRAF6^flox/flox^ mice with Lyz2- Cre mice. Genotyping was carried out after tail clipping on 4-week-old mice. Intratracheal instillations of BCG in female mice (18–22 g) were started when the mice were 8 weeks old.

### Cell lines

The RAW264.7 cell lines and HEK293T cell lines were obtained from the Shanghai Institute of Biochemistry and Cell Biology, Chinese Academy of Sciences (Shanghai, China), and the cells were cultured in Dulbecco’s Modified Eagle Medium (Gibco, California, USA) complemented with 10% fetal bovine serum (FBS, California, USA).

Bone marrow-derived Mφs (BMDMs) were harvested from 8- to 10-week-old TRAF6^flox/flox^ and TRAF6^flox/flox^; Lyz2^cre+/-^ cKO mice by flushing femurs and tibias with PBS. Cells were washed once with PBS and cultured in DMEM (Gibco, California, USA) supplemented with 10% FBS (Gibco, California, USA), 1% penicillin and streptomycin (Solarbio, Beijing, China), and 25 ng/mL colony-stimulating factor (MCE, California, USA) in non-tissue culture Petri dishes. The cells were then incubated at 37 °C for 7 days in culture dishes, and the media were replenished every 3 days. Adherent cells were washed and harvested with trypsin (Solarbio, Beijing, China).

### Mycobacterial cultures

Bacillus Calmette-Guerin (BCG) was purchased from the Centers of Disease Control and Prevention of China and preserved in the Key Lab of the Ministry of Education for Protection and Utilization of Special Biological Resources in Western China (Yinchuan, Ningxia). The Mtb *H37Rv* strains were obtained and cultured from the Fourth People’s Hospital of Ningxia Hui Autonomous Region (Yinchuan, Ningxia, China). The Mtb *H37Rv* and BCG were grown in Middlebrook 7H9 Broth supplemented with 10% OADC (BD Biosciences, 212240) at 37 °C with an atmosphere of 5% CO_2_. All experiments with Mtb *H37Rv* were performed in a Biosafety Level III containment lab of the Fourth People’s Hospital of Ningxia Hui Autonomous Region.

### Mice infection

Intratracheal instillation of PBS served as a negative control for BCG instillation. According to the random digital table method, TRAF6^flox/flox^ mice (*N* = 10) and TRAF6^flox/flox^; Lyz2^cre+/-^ mice (*N* = 10) were assigned to two groups and intratracheally injected with either control PBS or BCG (2 × 10^6^ CFU). The mice were euthanized at 21 days. The bronchoalveolar lavage fluid (BALF) and lung tissues were gathered for the recruitment of immune cells, the expression of autophagy-related proteins, CFU counting, and histopathological examination.

### Cells infection

Before infection, the bacterial suspensions were transferred into a 1.5 mL Eppendorf (EP) tube, and centrifuged, and the supernatant was discarded. The bacteria were washed and resuspended in 1× PBS and their concentration was estimated by turbidity measurements. The main concentration of bacteria was 1.5 × 10^8^ CFU/mL, which is equivalent to the McFarland 0.5 Turbidity Standard.

For Mtb *H37Rv* infection, RAW264.7 cells or BMDMs were seeded at 1 × 10^6^ cells per well and cultured overnight in DMEM complete culture medium, followed by infection with 10 MOI of Mtb *H37Rv*. After 6 h, cells were washed 3 times with 1× PBS and then cultured in fresh DMEM media for 2 days. For BCG infection, RAW264.7 cells or BMDMs were seeded in 6-well plates at a density of 1 × 10^6^ cells/well and cultured for 12 h before infection. The culture medium was replaced with fresh DMEM after BCG infection 6 h and the cells were collected at different time points to extract protein and RNA.

### Plasmid and siRNA transfection

TRAF6 wild-type plasmid and mutant plasmids were constructed according to TRAF6 sequence (NM_009424.3, CDS). These plasmids were conducted by General Biosystems (Anhui, China). HEK293T cells were transfected with plasmids using Lipofectamine^TM^ 3000 Transfection Reagent (Thermo Fisher Scientific, Massachusetts, USA) according to the manufacturer’s protocol. The empty vector plasmid (Mock) was used as negative control, and subsequent experimentation was performed after 48 h transfection.

ZETA LIFE Advanced reagent was used (Zeta-Life, San Francisco, USA) according to the manufacturer’s instructions. In brief, 1 × 10^6^ RAW264.7 cells (each well) were seeded in 6-well plates for 12 h. ZETA LIFE Advanced (10 μL) and siRNA duplexes (10 μL) were subsequently mixed while the plates were shaken gently. Three various *siRNA* targeting *TRAF6* (NM_009424.3, CDS) were conducted in GenePharma (Shanghai, China) and the sequences of the siRNAs are listed below.

#1153: Sense (5′-3′): GGAGAGUCGCCUAGUAAGATT

Antisense (5′-3′): UCUUACUAGGCGACUCUCCTT

#1810: Sense (5′-3′): GGUGUAGCGUCCAUGUACUTT

Antisense (5′-3′): AGUACAUGGACGCUACACCTT

#4524: Sense (5′-3′): GGCUGUUACCAUCUCUAGCTT

Antisense (5′-3′): GCUAGAGAUGGUAACAGCCTT

### Hematoxylin-eosin (H&E) staining

The mouse lung tissues were placed into 4% paraformaldehyde solution for 12 h (pH = 7.4) and embedded in paraffin. Sections of tissue samples were cut into 5–8 μm, sections and dewaxed. Next, stained with hematoxylin solution and eosin solution according to standard histological experimental procedures and then visualized using light microscopy (Olympus, Tokyo, Japan).

### Western blotting analysis

The lung tissues or different groups of RAW264.7 cells and BMDMs were lysed in RIPA lysis buffer containing a protease and phosphate inhibitor (Solarbio, Beijing, China). The protein concentration was confirmed by a BCA protein assay kit (Thermo Fisher Scientific, Massachusetts, USA). Moreover, the lysates were boiled for 5 min in 1× SDS sample buffer, and equal amounts of proteins from each sample were probed by SDS-PAGE. Then, the protein bands were transferred onto PVDF membranes. Membranes were blocked using 5% skim milk for 1 h at 37 °C and detected with the relevant primary antibodies and secondary horseradish peroxidase-conjugated antibodies. The proteins were visualized using ECL (Thermo Fisher Scientific, Massachusetts, USA) according to the manufacturer’s instructions. The details of the related antibodies are listed in the Antibodies and reagents section. For quantifications, densitometric analysis was done with ImageJ.

### Real-time PCR

Total RNA was isolated using TRIzol reagent (Invitrogen, California, USA) according to the manufacturer’s recommendation and reverse transcribed into cDNA using a PromeScript RT Kit (Takara, Osaka, Japan) as per the kit’s instructions. Relative gene expression was determined using a real‐time PCR Kit (ABclonal, Boston, USA) with QuantStudio 5 (Thermo Fisher Scientific, Massachusetts, USA) using the 2^‐ΔΔ Ct^ method, and β-actin was used for normalization. The correlating primers are the following sequences:

TRAF6 (Humo), forward: 5’ GGATTGTCCAAGGAGACAGGTT 3’, and reverse: 5’ AATTGGGGCTGTAGGGCAG 3’;

β-actin (Humo), forward: 5’ ACCGCGAGAAGATGACCCA 3’; and reverse: 5’ GGATAGCACAGCCTGGATAGCA 3’;

TRAF6 (Mouse), forward: 5’ CATGGACGCCAAACCAGAAC 3’; and reverse: 5’ CCCATGTCAAAGCGGGTAGA 3’;

β-actin (Mouse), forward: 5’ TGAGAGGGAAATCGTGCGTGACAT 3’; and reverse: 5’ ACCGCTCGTTGCCAATAGTGATGA 3’.

### Flow cytometry and cell sorting

To detect the infection rate of BCG-infected macrophages, BCG was stained with 5-Carboxyfluorescein diacetate N-succinimidyl ester (MedChemExpress, HY-D0056) and then infected at 5 MOI for 6 h in BMDMs. Then cells were digested with trypsin and resuspended with PBS for flow cytometry analysis as aforementioned.

Bronchoalveolar lavage fluid was collected by three lung lavages with 0.7 mL of PBS each using a blunt-end 21-gauge needle. For all experiments, cells were incubated in 0.5 μg Fc Block (BD Biosciences) for 10 min at RT. Surface staining was performed in the dark for 30 min at 4 °C in staining buffer. Cells were then washed twice with staining buffer followed by fixation in 1% paraformaldehyde solution. A comprehensive list of surface markers includes PE- conjugated anti‐F4/80 (0.2 mg/mL, eBioscience, 12-4801-82), FITC-conjugated anti‐CD11b (0.1 mg/mL, Proteintech, FITC-65055), PE-conjugated anti‐CD8 (0.1 mg/mL, Proteintech, PE-65069), FITC-conjugated anti‐ CD3 (0.1 mg/mL, Proteintech, FITC-65060), and PE-conjugated anti‐CD4 (0.1 mg/mL, Proteintech, PE-65141). For each sample, 50,000 cells were collected. Flow cytometry and data analysis were performed by using Sony MA900 (Sony Biotechnology, California, USA) and the FlowJo-V10 software.

### Bacterial loads

For measurement of the bacterial burden, the cells and mouse lung tissue were homogenized in phosphate-buffered saline with 0.5% Triton X-100 (MCE, New Jersey, USA), and serial dilutions of the homogenates were plated on duplicate plates of Middlebrook 7H10 agar at 37 °C. Bacterial colonies were counted after 21 days.

### Analysis of autophagic flux

The mRFP-GFP-LC3 adenovirus (Hanbio Biotechnology, Shanghai, China) was transfected into RAW264.7 cells to detect autophagic flux. Images were acquired using confocal fluorescence microscopy (LEICA, Germany). Yellow punctate described early autophagosomes, while red punctate indicated autolysosomes. Autophagic flux was calculated by the color change of GFP/mRFP. RAW264.7 cells were transfected with mRFP-GFP-LC3 (MOI = 30) for 48 h. Then, RAW264.7 cells transfected with *siRNA-NC* or *siRNA-TRAF6* were stimulated with BCG for 12 h.

### Transmission electron microscopy

RAW264.7 cells were incubated with BCG at an MOI of 10 for 12 h before being collected by centrifugation and fixed with electron microscope fixation solution. Then, the samples were rinsed with PBS 3 times every 15 min and dehydrated through graded ethanol (30, 50, 70, 80, 90, and 100%). The dehydrated cells were infiltrated and embedded with epoxy resin for 48 h sectioned (70 nm) and stained with uranyl acetate and lead citrate in preparation for observation using transmission electron microscopy (Tokyo, Japan). The data were quantified by counting the number of autophagosome-like structures per cross-sectioned cell by transmission electron microscopy.

### Autophagy rate

The autophagy rate was detected by Cyto-ID^®^ Autophagy Detection Kit (Enzo Life Sciences, Switzerland, USA). The culture solution was removed, and the cells were washed three times with PBS and collected in 1.5 mL tubes. After centrifugation (800 × g, 5 min), the cells were resuspended in 500 µL of dye buffer and incubated for 30 min at 37 °C, and a flow cytometry assay was performed.

### Bacteria staining

5-Carboxyfluorescein diacetate N-succinimidyl ester (MedChemExpress, HY-D0056) was used for bacteria staining. Briefly, the bacteria that grew to an OD_600_ of approximately 0.6–0.8 were centrifuged at 7000 × g for 5 min, and then the bacterial strains were washed three times with Hanks’ balanced salt solution (Solarbio) containing 0.05% Tween-80. The Hanks’ balanced salt solution containing 0.05% Tween-80 and dye was mixed at a ratio of 40:1 and stained at 37 °C for 30 min. The bacteria were then washed three times and finally resuspended in a DMEM medium containing 0.05% Tween-80 for infection.

### Immunofluorescence confocal microscopy

Briefly, cells were cultured on cell slides. After treatment, the cells were fixed with 4% paraformaldehyde for 30 min washed with PBS, and permeabilized with Triton X-100 solution (0.01% in PBS) 3 times. After blocking in 3% BSA for 1 h, the cells were incubated with primary antibodies diluted in 3% BSA overnight at 4 °C. Excess primary antibody was removed, the slides were washed with PBS and the samples were incubated with fluorescein-conjugated secondary antibodies (1:500) in PBS for 1 h at 37 °C in the dark. After the slides were washed with PBS, they were mounted with a DAPI-containing mounting medium (ZSGB-BIO, Beijing, China). Images were obtained using a laser confocal microscope (LEICA, Germany). For the co‐localization of Rab7 and TRAF6 on RAW264.7 cells/BMDMs, the rabbit monoclonal anti-Rab7 (1:100, Cell Signaling Technology, 9367) was mixed with mouse monoclonal anti-TRAF6 (1:100, Santa Cruz Biotechnology, SC-8409). For the co‐localization of bacteria and LC3 on RAW264.7 cells/BMDMs, an anti-LC3 antibody (1:100, Proteintech, 14600-1-AP) was used. For the co‐localization of HA-Rab7 and STX17 on HEK293T cells, the mouse monoclonal anti-HA (1:100, Proteintech, 66006-2-Ig) was mixed with rabbit polyclonal anti-STX17 (1:100, Proteintech, 17815-1-AP). For the colocalization of FLAG-TRAF6 and HA-Rab7, the mouse monoclonal anti-HA (1:100, Proteintech, 66006-2-Ig) and the rabbit monoclonal anti-FLAG (1:100, Cell Signaling Technology, 14793) were performed. For the detection of EEA1, a rabbit monoclonal antibody was used (1:100, Abcam, 109110). Fluorescence intensity was analyzed by using ImageJ software. The colocalization analysis of immunofluorescence images was performed with ImageJ software using the colocalization plugin Coloc 2 (https://imagej.net/Coloc_2).

### Co-immunoprecipitation

For the Co-immunoprecipitation (Co-IP) assay, cells were lysed in CHAPS buffer containing protease/phosphatase inhibitor cocktail tablets for 20 min. After centrifugation at 12,000 × g for 15 min at 4 °C, 40 µL of cleared lysate were used for western blotting analysis, while the remaining lysates were used to perform IP reactions with 2 µg primary antibodies overnight at 4 °C. The following day, the lysate was supplemented with protein A/G PLUS-agarose for 4 h at 4 °C. Immune complexes were eluted in 100 µL of 1× loading buffer and analyzed by western blotting. For endogenous immunoprecipitation assay, RAW264.7 cells or BMDMs were infected with BCG. Immunoprecipitation assay was performed using IgG (Cell Signaling Technology, 7076), anti-TRAF6 (Santa Cruz Biotechnology, sc-8409), or anti-Rab7 (Santa Cruz Biotechnology, sc-8409, sc-376362) antibody. Immunoblot (IB) assay was performed with anti-TRAF6 (Abcam, 40675), anti-Rab7 (Cell Signaling Technology, 9367), STX17 (Proteintech, 17815-1-AP) and anti-Ub (Cell Signaling Technology, 3936) antibodies. In the exogenous experiment, the plasmids were transfected into HEK293T cells using Lipofectamine^TM^ 3000 for 48 h. The cell lysates were immunoprecipitated with either anti-FLAG (Proteintech, 20543-1-AP), or anti-HA (Cell Signaling Technology, 3724) antibody. Immunoprecipitated complexes were separated by 10% SDS-PAGE and detected with anti-FLAG (Proteintech, 20543-1-AP), or anti-HA (Cell Signaling Technology, 3724), or anti-MYC (Proteintech, 60003-2-Ig). The quantitative analysis method refers to the article of Burckhardt et al. [81, 82].

### Statistical analysis

The data statistical analysis was performed using the GraphPad Prism version (GraphPad Software, USA). Unless otherwise specified, the data are presented as the mean ± SEM. The data were statistically evaluated to compare differences between different groups by one-way ANOVA; *p < 0.05; **p < 0.01; ***p < 0.001.

### Supplementary information

Supplementary material

## Data Availability

The datasets used and/or analyzed during the current study are available from the corresponding author upon reasonable request.
