# Supplementary material for: TRAF6 triggers Mycobacterium-infected host autophagy through Rab7 ubiquitination
Source: Cell Death Discov. 2023 Nov 28;9:427. doi: 10.1038/s41420-023-01731-4 (PMC10684575; doi:10.1038/s41420-023-01731-4)
Supplement: Supplementary file 1 — Supplementary material [file 41420_2023_1731_MOESM1_ESM.pdf]

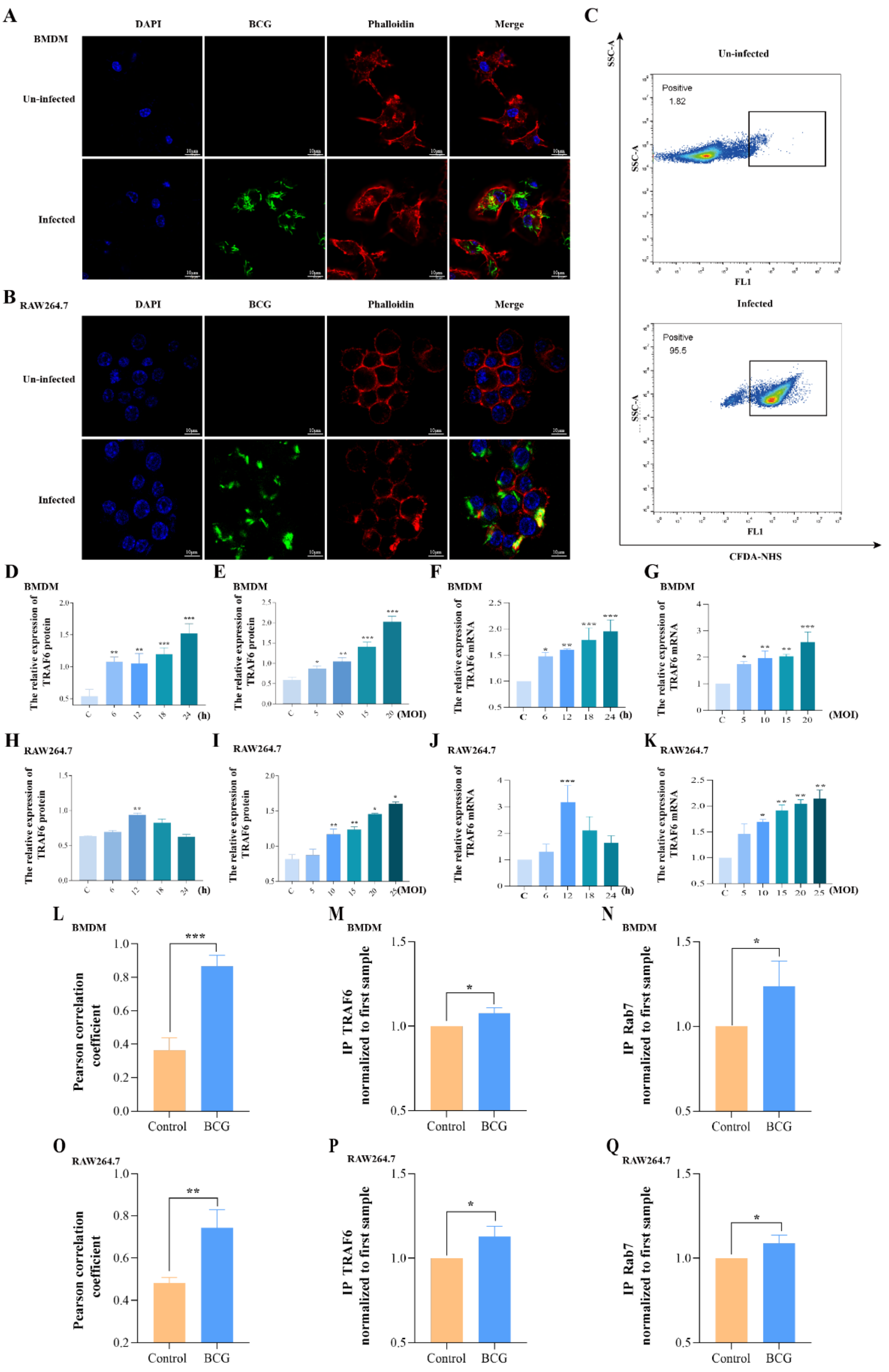

Figure S1. *Mycobacterium* infection elevates TRAF6 expression and enhances the binding of TRAF6 and Rab7. (**A-B**) Red fluorescence represents phalloidin, green fluorescence represents BCG, and blue fluorescence represents cell nucleus. Scale bar:

10 µm. BMDMs (**A**), RAW264.7 (**B**). (**C**) BMDMs were used as recipient cells for infection; BCG infection efficiency was determined by checking CFDA expression by flow cytometry. (**D**) (**F**) Western blotting (**D**) and RT-PCR (**F**) analysis of TRAF6 in BMDMs infected with 5 MOI BCG for 6, 12, 18, and 24 h respectively. (**E**) (**G**) Western blotting (**E**) and RT-PCR (**G**) analysis of TRAF6 expression in BMDMs infected with 5, 10, 15, and 20 MOI BCG for 6 h respectively. (**H**) (**J**) Western blotting (**H**) and RT-PCR (**J**) analysis of TRAF6 in RAW264.7 cells infected with 10 MOI BCG for 6, 12, 18, and 24 h respectively. (**I**) (**K**)Western blotting (**I**) and RT-PCR (**K**) analysis of TRAF6 expression in RAW264.7 cells infected with 5, 10, 15, 20, and 25 MOI BCG for 12 h respectively. (**L**) Colocalization analysis of TRAF6 and Rab7 in BCG-infected BMDMs. (**M-N**) Relative intensity of the TRAF6 (**M**) and Rab7 (**N**) Co-IP bands were semi-quantitative analyzed in BMDMs. (**O**) Colocalization analysis of TRAF6 and Rab7 in BCG-infected RAW264.7 cells. (**P-Q**) Relative intensity of the TRAF6 (**P**) and Rab7 (**Q**) Co-IP bands were semi-quantitative analyzed in RAW264.7 cells. The protein ratio was calculated by ImageJ densitometry analysis. The colocalization coefficients were expressed as Pearson correlation coefficient. The semi-quantitative analysis method of Co-IP refers to the article of Burckhardt et al.[83, 84]. Data were shown as the mean ± SEM, and one representative experiment from three independent experiments is shown. *p <0.05; **p < 0.01; ***p < 0.001.

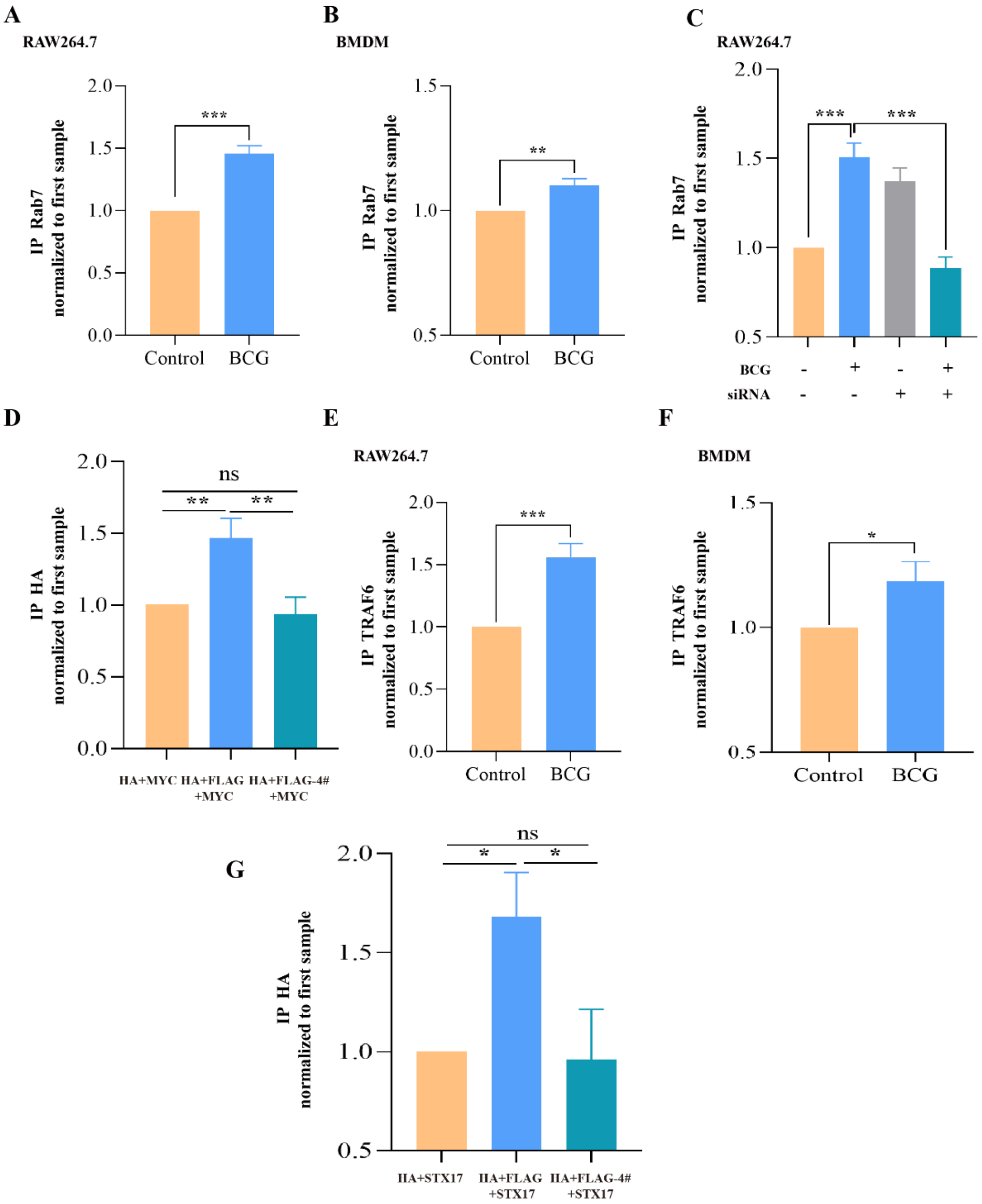

Figure S2. TRAF6 strengthens the binding ability between Rab7 and STX17 by promoting Rab7 ubiquitination in *Mycobacterium*-infected macrophages. (**A**) Co-IP analysis of the ubiquitinated Rab7 protein levels in BCG-infected RAW264.7 cells. (**B**) Co-IP analysis of the ubiquitinated Rab7 protein levels in BCG-infected BMDMs. (**C**) In BCG-infected BMDMs, Rab7 was precipitated (IP: Rab7), and the ubiquitylation status of Rab7 was analyzed by Western blotting when TRAF6 knockdown. (**D**) The

status of Rab7 ubiquitination was assessed by performing IP followed by Western blotting for ubiquitin in HEK293T cells. (**E**) Co-IP with anti-TRAF6 for IP, and anti-TRAF6 and anti-STX17 for IB was performed in RAW264.7 cells. (**F**) Co-IP with anti-TRAF6 for IP, and anti-TRAF6 and anti-STX17 for IB was performed in BMDMs. (**G**) Membrane fraction was analyzed for HA-Rab7/STX17 binding by IP/IB. The protein ratio was calculated by ImageJ densitometry analysis. The semi-quantitative analysis method of Co-IP refers to the article of Burckhardt et al. [83, 84]. Data were shown as the mean ± SEM, and one representative experiment from three independent experiments is shown. **p* <0.05; ***p* < 0.01; ****p* < 0.001.

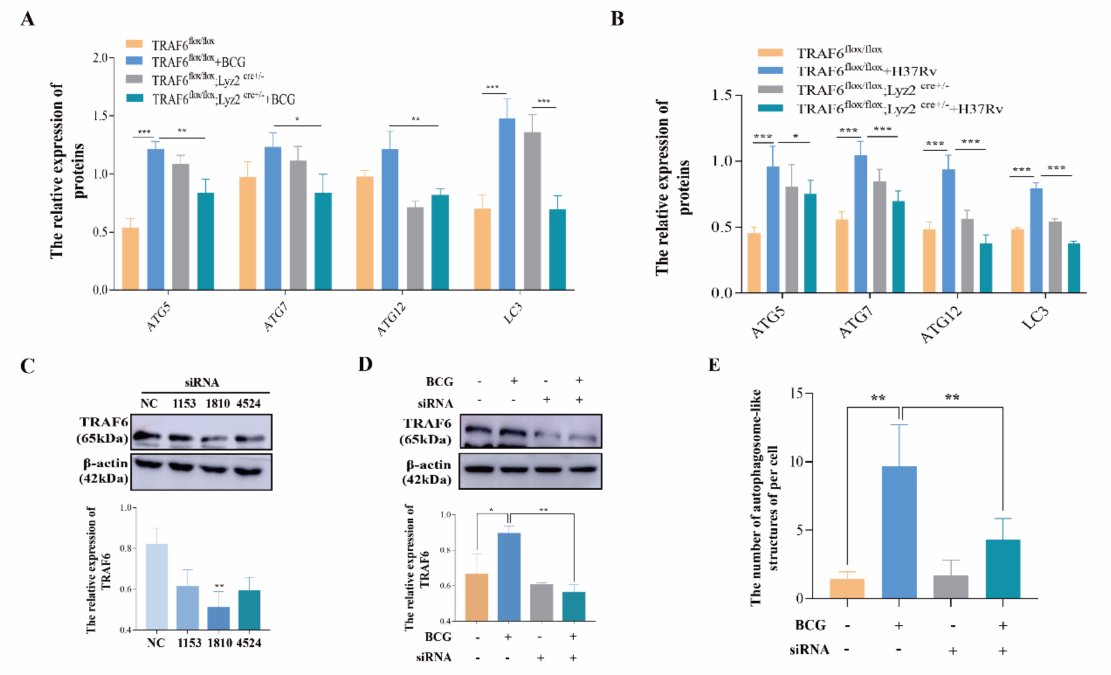

Figure S3. TRAF6 contributes to *Mycobacterium*-mediated autophagosome initiation and formation. (**A**) The expression of ATG5, ATG7, ATG12 and LC3 in BCG-infected BMDMs. (**B**) The expression of ATG5, ATG7, ATG12 and LC3 in *H37Rv*-infected BMDMs. (**C**) To avoid an off-target effect, three kinds of siRNAs were selected for transfection and to detect silence efficiency. The expression of TRAF6 was observed

by Western blotting analysis. (**D**) RAW264.7 macrophages were transfected with *siRNA-NC* or *siRNA-1810* for 36 h and then infected with BCG for 12 h. The expression of TRAF6 was assayed in the transfected macrophages by Western blotting analysis. (**E**) Quantification of the number of autophagosome-like structures, reported as the average number of autophagosome-like structures per cell. The protein ratio was calculated by ImageJ densitometry analysis. Data were shown as the mean ± SEM, and one representative experiment from three independent experiments is shown. *$p < 0.05$; **$p < 0.01$; ***$p < 0.001$.
